# Complete Chloroplast Genome Sequence of *Triosteum sinuatum*, Insights into Comparative Chloroplast Genomics, Divergence Time Estimation and Phylogenetic Relationships among Dipsacales

**DOI:** 10.3390/genes13050933

**Published:** 2022-05-23

**Authors:** HaiRui Liu, WenHui Liu, Israr Ahmad, QingMeng Xiao, XuMin Li, DeJun Zhang, Jie Fang, GuoFan Zhang, Bin Xu, QingBo Gao, ShiLong Chen

**Affiliations:** 1 State Key Laboratory of Plateau Ecology and Agriculture, Qinghai University, Xining 810008, China; 2018990018@qhu.edu.cn (H.L.); djzhang@qhu.edu.cn (D.Z.); 2 College of Eco-Environmental Engineering, Qinghai University, Xining 810008, China; 1800601045@qhu.edu.cn (Q.X.); y200713000501@qhu.edu.cn (X.L.); 1800601039@qhu.edu.cn (J.F.); 190710020230@qhu.edu.cn (G.Z.); 190710020235@qhu.edu.cn (B.X.); 3 Key Laboratory of Adaptation and Evolution of Plateau Biota, Northwest Institute of Plateau Biology, Chinese Academy of Sciences, Xining 810001, China; slchen@nwipb.cas.cn; 4 Department of Geological Engineering, Qinghai University, Xining 810016, China; liuwenhui222@lzb.ac.cn; 5 Department of Botany, Women University of AJK, Bagh 12500, Pakistan; driabotany@wuajk.edu.pk

**Keywords:** *Triosteum* L., chloroplast genome, Dipsacales, phylogeny

## Abstract

*Triosteum himalayanum*, *Triosteum pinnatifidum* (*Triosteum* L., Caprifoliaceae, Dipsacales) are widely distributed in China while *Triosteum sinuatum* mainly occurrs in northeast China. Few reports have been determined on the genus *Triosteum*. In the present research, we sequenced 2 chloroplast genomes of *Triosteum* and analyzed 18 chloroplast genomes, trying to explore the sequence variations and phylogeny of genus *Triosteum* in the order Dipsacales. The chloroplast genomes of the genus *Triosteum* ranged from 154,579 bp to 157,178 bp, consisting of 132 genes (86 protein-coding genes, 38 transfer RNA genes, and 8 ribosomal RNA genes). Comparative analyses and phylogenetic analysis supported the division of Dipsacales into two clades, Adoxaceae and six other families. Among the six families, a clade of Valerianaceae+Dipsacaceae was recovered as a sister to a clade of Morinaceae+Linnaeaceae. A closer relationship of *T. himalayanum* and *T. pinnatifidum* among three species was revealed. Our research supported that *Lonicera* *ferdinandi* and *Triosteum* was closely related. *Zabelia* had a closer relationship with *Linnaea borealis* and *Dipelta* than Morinaceae. The divergence between *T. sinuatum* and two other species in *Triosteum* was dated to 13.4 mya.

## 1. Introduction

Chloroplasts (cp) are involved in photosynthesis and provide energy for green plants [1,2]. The double-stranded and circular chloroplast genome is composed of two short inverted repeat regions (IRa and IRb) which are separated by a large single copy (LSC) region and a small single copy (SSC) region [3,4,5]. The sequence of chloroplast genome is highly conserved and similar in the order of Dipsacales. Due to its short length, conserved structure and type of genes, together with few repeat regions, the complete chloroplast genome can provide abundant information for species phylogeny and taxonomy. Hence, the complete chloroplast genome is widely used in plant phylogeny and evolution study [6,7,8,9,10].

*Triosteum* Linn. is perennial medicinal herb, belonging to family Caprifoliaceae (Dipsacales). Some of the *Triosteum* species can be used as medicine. Previously, several study were conducted to determine the phylogeny and evolutionary lineage in Dipsacales [4,7,11,12,13,14]. The order Dipsacales is widely considered to consist of Caprifoliaceae s.l. and Adoxaceae according to the APG IV and some other previous studies [11,12,13]. Diervillaceae, Dipsacaceae, Linnaeaceae, Morinaceae, Caprifolieae and Valerianaceae belong to the large clade Caprifoliaceae s.l., comprising 41 genera (including *Acanthocalyx*, *Dipsacus*, *Patrinia*, *Linnaea*, *Weigela*, *Triosteum*, *Morina*, *Lonicera*, *Triplostegia*, etc.). Additionally, Adoxaceae includes five genera (*Adoxa*, *Sambucus*, *Sinadoxa*, *Tetradoxa and Viburnum*).

However, the phylogentic evolution of *Triosteum* within Dipsacales was poorly invested. In this study, we focused on three species of the genus *Triosteum* L., including *T. himalayanum*, *T. pinnatifidum*, *T. sinuatum*. These perennial herbs are widely distributed in China as well in Japan and Russia (*T. pinnatifidum*, *T. sinuatum*) [15,16]. Studies of chemical constituents have been conducted on *T. himalayanum* and *T. pinnatifidum.* Additionally, few evolutionary relationships between *Triosteum* and other genera have been revealed [17,18,19,20]. Gould and Donoghue (2000) used sequences of ITS and GBSSI to reveal the biogeographic pattern of *T. himalayanum* and *T. pinnatifidum* in China and *T. sinuatum* in Japan. According to the results of the previous studies, *Triosteum* was close to *Lonicera* in order Dipsacales. Based on the phylogeographic structure of *T. himalayanum* localities, the phylogeny of haplotypes and palaeodistributional reconstruction, both westwards and northwards expansion of this species was postulated [15]. For *T. pinnatifidum*, the genetic structure, unimodal mismatch distribution, star-like network, together with demographic history analysis supported the “dispersal into the Qinghai-Tibetan Plateau” hypothesis [21].

Here, the whole chloroplast (cp) genome sequences of *T. pinnatifidum*, *T. sinuatum* and *Linnaea borealis* were assembled and annotated. The cp genome of *T. sinuatum* was utilized for the first time for the phylogenetic relationship and tracing of evolutionary lineage in *Triosteum*. A total of 20 chloroplast genome sequences of Dipsacales were generated, and six sequences from Apiales and Lamiales were used as outgroup taxa. Seventeen cp genome sequences in Dipsacales were downloaded from NCBI database. The sequence variations and detailed interrelationship of 20 species in the order Dipsacales were explored. The present study was carried out to identify the variation of hotspot regions in chloroplast genomes, analyze the simple sequence repeat (SSR) diversity, and calculate relative synonymous codon usage (RSCU) frequency. The phylogenetic relationship between *Triosteum* and other lineages in the order Dipsacales was reconstructed, and divergence time was estimated based on cp genome.

## 2. Materials and Methods

### 2.1. Sampling, DNA Isolation, Sequencing and Assembly

Fresh leaves of *T. pinnatifidum* were collected in Tibet. Leaves of *Linnaea borealis* and *T. sinuatum* were collected in Jilin Province. All the specimens were tagged, dried, poisoned and pasted on standard herbarium sheets. The voucher specimens are deposited in the Herbarium of Northwest Institute of Plateau Biology (HNWP), Xining, Qinghai, China for future reference. The genomic DNA of *T. pinnatifidum* was extracted from fresh leaves using the cetyltrimethyl ammonium bromide (CTAB) method (Doyle, 1987) with some modifications [22]. The Illumina sequencing of *T. sinuatum* and *L. borealis* was carried out by the Genepioneer Biotechnologies (Nanjing, China). Assembly was performed with SPAdes v3.10.1, SPACE v2.0 and Gapfiller v2.1.1 [23]. We rearrange the corrected pseudo genome in order and obtained a complete circular sequence. In addition, 23 complete sequences of chloroplast genomes were selected and downloaded from GeneBank after sequences check, comprising 17 of Dipsacales, 3 of Apiales and 3 of Lamiales (Table 1).

### 2.2. Genome Annotation

We annotated the genome sequence using GeSeq (https://chlorobox.mpimp-golm.mpg.de/geseq.html (accessed on 10 January 2021)) and used the available sequences downloaded from GeneBank as a reference [24]. The manual correction of annotation was performed with Sequin v.15.50 and ApE [25]. The complete chloroplast genome sequences were submitted to GeneBank. Circular plastid genome maps were obtained from OGDRAW (https://chlorobox.mpimp-golm.mpg.de/OGDraw.html (accessed on 12 January 2021)) [26].

### 2.3. Genome Structure and Comparative Analysis

Structure and gene contents among the plastomes were manually compared with Notepad++ v7.5.9. Alignments of 20 complete chloroplast genome sequences were visually compared using the Shuffle-LAGAN Model of mVISTA [27], which uses *T. sinuatum* as a reference. Boundaries between IR and SC regions of these species were also visualized with IRscope (https://irscope.shinyapps.io/irapp/ (accessed on 14 April 2021)) [28].

### 2.4. Distribution of Simple Sequence Repeats (SSRs)

Chloroplast simple sequence repeat (cpSSR) was widely used to study the phylogeny of plants and genetic structure for its high variability. MISA_web (https://webblast.ipk-gatersleben.de/misa/ (accessed on 24 January 2021)) was used to detect the SSRs in *T. sinuatum* with the following parameters: 10 repeat units for mononucleotide SSRs, 5 repeat units for dinucleotide SSRs, 4 repeat units each for trinucleotide, and 3 repeat units each for tetra-, penta-, and hexanucleotide SSRs [29].

### 2.5. Codon Usage Pattern

We used the CodonW software (John Peden, http://www.molbiol.ox.ac.uk/cu (accessed on 24 May 2021), version 1.4.2) to calculate the number of amino acid codon in chloroplast genomes of these twenty species [30]. The FASTA files containing of 20 chloroplast genome sequences were downloaded from NCBI before RSCU analysis. The results were recorded by column graph with R language after running program.

### 2.6. Phylogenetic Analysis

To reveal the phylogenetic position of the genus *Triosteum*, a total of 26 species were selected. Out of 26 species, the ingroup contains 20 species that belong to the order Dipsacales. Clustalw v2.1 was used for genome alignment of all species. Iqtree v1.6.12 was used to find the best substitution model and constructed maximum likelihood (ML) tree with the use of GTR+F+G4 model [31,32]. The visualization of the ML tree was completed with Figtree v1.4.4 (http://tree.bio.ed.ac.uk/software/fgtree (accessed on 23 March 2022)).

### 2.7. Divergence Time Estimation

BEAST v1.8.0 was used to estimate the divergence time of the genus *Triosteum*, the and GTP+G+I model was chosen as a substitution model [33]. The analysis was calculated on the basis of two fossils of the genus *Dipelta* and the genus *Viburnum*. Tree prior was determined as the Yule process and the clock model was a lognormal relaxed clock model. The three calibration points were the genus *Dipelta* with lognormal prior (mean = 36 Ma, SD = 1.0), the genus *Viburnum* with lognormal prior (mean = 89.3 Ma, SD = 1.0) and the order Dipsacales with normal prior (mean = 103 Ma, SD = 1.0). A total of 10,000,000 generations were run, and the Tracer was used to determine the correctness. Finally, with 25% burn-in, a maximum clade credibility tree was conducted using TreeAnnotator [33].

## 3. Results and Discussion

### 3.1. Plastome Features and Gene Content

**Figure 1:**
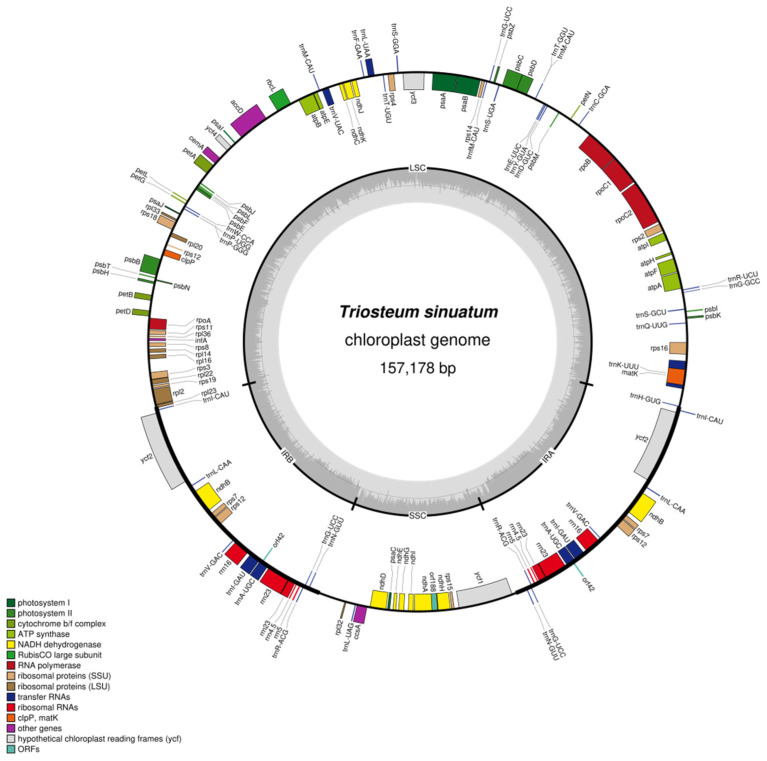
Gene map of the *Triosteum sinuatum* chloroplast genome. Genes on the inside of the circle are transcribed in the clockwise direction, and genes outside the circle are transcribed in the counterclockwise directions. Genes with different functions are shown in different color blocks. The inverted repeat (IRa and IRb) regions are indicated by the thick lines. LSC and SSC indicate large and small single copy, respectively.

Genome maps of *T. sinuatum* in Dipsacales were drawn with ODDRAW (Figure 1). The chloroplast genome showed a typical double-stranded circular structure, length of *T. himalayanum*, *T. pinnatifidum* and *T. sinuatum* was 154,579 bp, 154,896 bp and 157,178 bp, respectively. Each chloroplast genome was composed of a large single copy region (89,007–90,758 bp) and a small single copy region (18,656–120,543 bp) separated by two inverted repeat regions (22,673–23,882 bp). Protein-coding genes *orf42*, *orf188* and tRNA gene *trnP*-*GGG* were lost in *T. pinnatifidum*. In particular, *trnP-UGG* could not be found in *T. himalayanum*.

Twenty complete chloroplast genomes were different in lengths, ranging from 151,267 bp (*Patrinia scabra*) to 161,576 bp (*Linnaea borealis*) (Table 1). Except for *Linnaea borealis,* the lengths of the other 19 sequences were less than 160 kb, which mainly reflected in the LSC and IR regions. The LSC length in Dipsacales ranged from 86,340 bp (*Adoxa moschatellina*) to 90,750 bp (*Acanthocalyx alba*), and the IR region ranged from 22,673 bp (*T*. *pinnatifidum*) to 29,210 bp (*Linnaea borealis*). GC contents of the complete chloroplast genomes are similar, which are approximately 38%. The structure characteristics are similar to the chloroplast genomes of most angiosperms [34,35,36]. Most of plastomes encoded about 132 genes, including 86 protein coding genes, 38 tRNA genes, and 8 rRNA genes (Table 2). *lhb*A only existed as a protein coding gene in Adoxaceae.

Nine out of 86 protein coding genes were for large subunits of ribosome (*rpl33*, *rpl20*, *rpl36*, *rpl14*, *rpl16*, *rpl22*, *rpl2*, *rpl23*, *rpl32*), 12 were for small subunits of ribosome (*rps16*, *rps2*, *rps14*, *rps4*, *rps18*, *rps12*, *rps11*, *rps8*, *rps3*, *rps19*, *rps7*, *rps15*), 4 were for RNA polymerase (*rpoC2*, *rpoC1*, *rpoB*, *rpoA*), 20 were for photosystem (*psaB*, *psaA*, *psaI*, *psaJ*, *psaC*, *psbA*, *psbK*, *psbI*, *psbM*, *psbD*, *psbC*, *psbZ*, *psbJ*, *psbL*, *psbF*, *psbE*, *psbB*, *psbT*, *psbN*, *psbH*) and 6 were for ATP synthase (*atpA*, *atpFa*, *atpH*, *atpI, atpE*, *atpB*). Among all these genes, 13 genes contained one intron, including 9 protein coding genes (*atpF*, *ndhA*, *ndhB*, *petB*, *petD*, *rpl16*, *rpl2*, *rpoC1*, *rps16*) and 4 tRNA genes, while one gene (*rps12*) contained two introns (Table 2). Fifteen genes (all four rRNAs genes, seven tRNAs genes, and four protein coding genes) were duplicated in the IR regions (Table 2).

### 3.2. Comparative Analyses

The sequence of *T. sinuatum* was used as a reference to assess similarity and differences among 20 species of Dipsacales. The alignment of sequences was visualized with an mVISTA plot (Figure S1). Alignments of 20 species showed that the whole chloroplast genome is highly conserved. The sequences in the single copy regions were more divergent than those in inverted repeat regions. In addition, more variation was found in non-coding regions than in coding regions. The results of the present study are in accordance with previous research by Cheng et al., 2020, Alzahrani et al., 2021 and Wang et al., 2021. [34,37,38]. The sequences of *T. sinuatum*, *T. himalayanum* and *T. pinnatifidum* showed high similarity but exhibited marked differences in the 133k region and the region of *accD*. There was greater differentiation where five plastid genomes lost the gene *ndhF* (*Adoxa moschatellina*, *Sinadoxa corydalifolia*, *Sambucus williamsii*, *Viburnum odoratissimum* and *Viburnum dilatatum*). This may considered be as evidence that the genera *Sambucus and Viburnum* should be placed in Adoxaceae from Caprifoliaceae.

**Figure 2:**
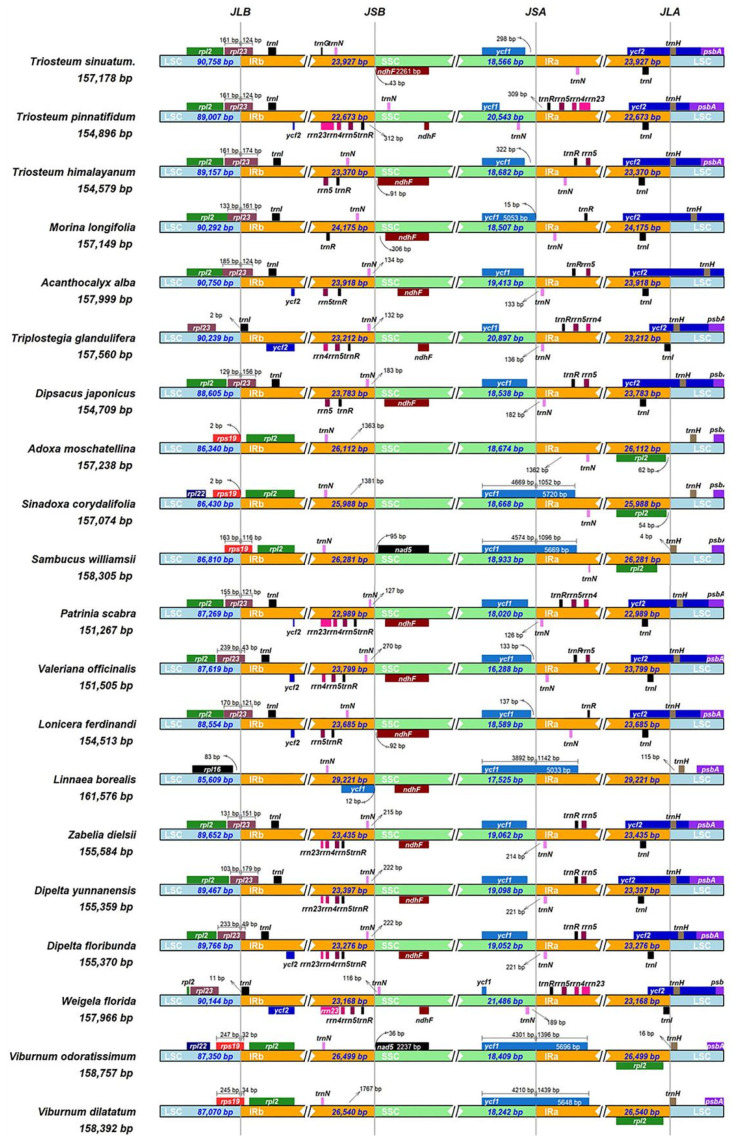
Comparisons of LSC, SSC and IR region boundaries among the chloroplast genomes of 20 species in Dipsacales.

The sizes of chloroplast genomes in Dipsacales species are similar but different for the main factors, the expansion and contraction of inverted repeat regions [39,40,41]. The border structure of 19 plastid genomes in the order Dipsacales was compared to analyse how expansion and contraction in IR regions affect the length of chloroplast genome. Detailed comparisons of LSC, SSC and inverted repeat regions are shown in Figure 2. The *rpl23* gene of 12 species was located in IRb, extending into the LSC region by about 43–179 bp. By contrast, for *Weigela florida* and *Triplostegia glandulifera*, the *rpl23* gene was found in the LSC region. Additionally, the IR/SC boundary in three plastomes (*Sambucus williamsii*, *Viburnum dilatatum* and *Viburnum odoratissimum*) was located on *rps19*. The *trnN* gene of 18 species excluding *T. pinnatifidum* and *Weigela florida* resided in the IRb and IRa region. In addition, the *ndhF* gene was positioned within the SSC region similarly. The *nad5* gene appeared only on the SSC region of *Sambucus williamsii* and *Viburnum odoratissimum*, which was 36 bp and 95 bp from the boundary between the IRb/SSC. On the other hand, *ycf2* was mainly found in the IRa/LSC region, while the *trnH* gene was inserted into the fragment distributed in the LSC region. The *ycf1* genes of 14 species are mainly located in the SSC region. However, in *Linnaea borealis* and the family Adoxaceae (except *Adoxa moschatellina*), the *ycf1* gene expanded to IRa region for 5033 bp to 5730 bp.

### 3.3. SSR Analysis of Triosteum sinuatum *Maxim.* Chloroplast Genomes

Chloroplast simple sequence repeats (SSRs) are crucial in phylogenetic analyses and are considered to be useful in identification of species [42,43,44]. Numerous SSRs were detected in 26 chloroplast genome sequences and their amounts ranged from 36 to 77. In total, 76 SSRs of six types (mononucleotide, dinucleotide, trinucleotide, tetranucleotide, pentanucleotide and hexanucleotide repeats) were found in *T. sinuatum*. The most plentiful type of SSRs was mononucleotide repeats with A/T types (63.2%), followed by dinucleotide (10.5%), tetranucleotide (10.5%), trinucleotide (7.9%), pentanucleotide (5.3%), hexanucleotide (2.6%) repeats (Figure S2a). Mononucleotide repeats, which were largest in number, consisted of A (41.7%) and T (58.3%) motifs, while dinucleotide repeats, which were second largest in number, consisted of AT (37.5%) and TA (62.5%) motifs (Figure S2b). Our results match with many previous studies [39,45,46]. Further analysis showed that the most SSRs were detected in the LSC region (77.6%), with a small portion located in the SSC (14.5%) and IR (7.9%) regions (Figure S2b). In addition, 80.2%, 13.2%, and 6.6% were, respectively, distributed in the intergenic spacer (IGS), protein coding genes (CDS) and introns (Figure S2d). SSRs detected in our study will contribute to providing data for molecular marker development in future evolutionary studies.

### 3.4. Codon Usage Pattern

Codon usage preference is a common phenomenon in nature, mainly determined by the dynamic balance of gene mutation and natural selection [47]. In addition, it is also related to gene coding structure and function, gene expression and other factors in the evolution process [37]. Natural selection usually causes organisms to prefer to use optimal codons, and mutation will lead to the existence of some non-optional codons. Different species or different genes of the same species have different preferences for codon use after long-term evolution [48,49]. The codon W was used to calculate the relative synonymous codon usage (RSCU) of 20 species. Additionally, the frequency of used codons for amino acids is presented in Figure S3. A codon with an RSCU value more than 1 (RSCU > 1) was considered as a preferred codon because its investigated usage frequency is more than the expected one [50]. AGA is the most frequently used codon (RSCU > 1) in the chloroplast genomes among 20 species. Meanwhile, the lowest frequency codon is CGC (RSCU < 0.51), which encodes the same amino acid (Arginine) as AGA. There is difference among 20 species, but high conservatism was showed in codon usage. In addition, the analysis of the preference of target gene and receptor genomic codon by bioinformatics is an important supplement to phylogenetic analysis.

### 3.5. Phylogenetic Relationships

**Figure 3:**
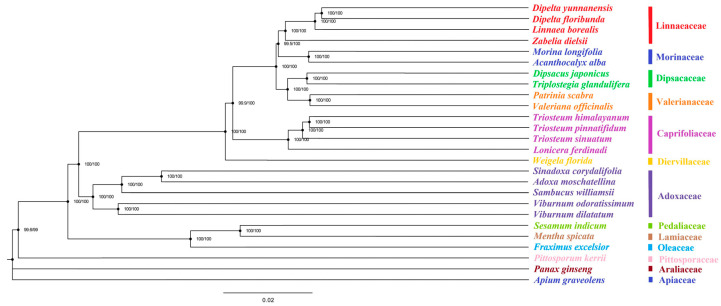
ML tree of 26 species. Twenty species are within Dipsacales.

The phylogenetic relationship between *Triosteum* and other genera in the order Dipsacales was constructed on the base of cp genomes of 20 ingroup and 6 outgroup species (Apiales and Lamiales) using Maximum likelihood method (Figure 3). The current study presents highly resolved phylogenies of Dipsacales based on complete plastome sampling of all families in Dipsacales. The present study strongly supports the previous research which also divided 20 Dipsacales species into two caldes [4,7,51,52,53]. One clade is Adoxaceae, and the other clade consisted of six major lineages with a highly supported topology of (Diervillaceae, (Caprifoliaceae, ((Valerianaceae, Dipsacaceae), (Morinaceae, Linnaeaceae)))). Moreover, our results indicate that the family Dipsacaceae (*Triplostegia glandulifera*, *Dipsacus japonicus*) is a sister to the family Valerianaceae. The family Morinaceae was placed as a sister to the family Linnaeaceae. However, in our study, *Zabelia dielsii* showed close relationships with *Linnaea* and *Dipelta* with high support rather than with Morinaceae, which is in conflict with a previous study [4,51]. According to the ML tree, three species of *Triosteum* clustered in one clade with a 100% bootstrap value. Among the three species of *Triosteum*, *T. pinnatifidum* and *T. himalayanum* are supposed to have a closer relationship. The topology result was consistent with the studies of Donoghue et al. (2003) and Liu et al. (2019) [11,54]. Here, complete chloroplast genome provided a stronger support. *Lonicera ferdinandi* and the three species in the genus *Triosteum* clustered into a clade, suggesting a very close phylogenetic relationship between the genus *Lonicera* and the genus *Triosteum*. Phylogenetic analysis in our study served as a baseline for further phylogeny studies on *Triosteum* and Dipsacales.

### 3.6. Divergence Time Estimation

**Figure 4:**
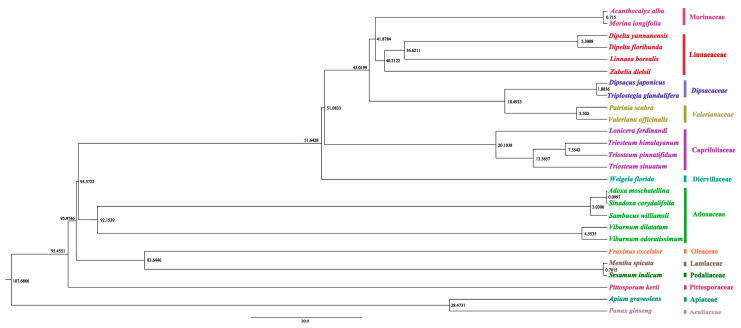
Divergence times analysis based on the chloroplast plastome.

The result of divergence time estimation of 26 species based on chloroplast sequences is shown in Figure 4. The divergence times estimated in our study are more recent than those in previous study by Fan et al. (2018) and Wang et al. (2019) [4,7]. The age of the crown node of Dipsacales was estimated to be upper Cretaceous, approximately 95.6 million years ago (mya). Morinaceae, Dipsacaceae, Valerianaceae, Linnaeaceae and Caprifoliaceae separated from Diervillaceae at about 51.6 mya. The separation between Caprifoliaceae and the clade Morinaceae+Dipsacaceae+Valerianaceae+Linnaeaceae occurred at 51.1 mya. The divergence between Morinaceae and Linnaeaceae was dated to 41.9 mya in the Eocene, which was later than that of the previous study by Wang et al. (2019, in the upper Cretaceous) [4]. These differences between current and previous studies are likely due to the influence of the amount of data and species sampling. The speciation of most of the species investigated here occurred after the beginning of Miocene. *Triosteum* and *Lonicera* separated at the time 20.1 mya. The divergence between *T. sinuatum* and other two *Triosteum species* was dated to 13.4 mya. The divergence between *T*. *himalayanum* and *T*. *pinnatifidum* was dated back to 7.6 mya, suggesting that these two species experienced divergence for a long time.

## 4. Conclusions

In this study, we reported the complete chloroplast genome of *Triosteum* of the order Dipsacales based on the Illumina platform. The full length of three species in *Triosteum* ranged from 154,513 to 157,178 bp. For 20 species in the order Dipsacales, our study investigated the variation of sequences and SSRs, compared genome structure, evaluated Codon usage bias, reconstructed phylogenetic relationships and estimated divergence times. A high similarity of sequences was shown within 20 Dipsacales species, but sequences were significantly different in 133k region and region of *accD*. Within Dipsacales, two major clades were clearly defined: Adoxaceae and another six families with high support. *T*. *pinnatifidum* and *T. himalayanum* are supposed to have a closer relationship than *T. sinuatum* in the genus. The divergence between *T. sinuatum* and other two *Triosteum* species was dated to 13.4 mya. The diversification of Dipsacales occurred at about 95.6 mya in the Cretaceous. The present research is the first documented report in China that assembled the choloplast sequence of *T*. *sinuatum*, contributing to further study. We provided stronger support for future studies on molecular identification and the better understanding of phylogeny in *Triosteum* and Dipsacales.

## Figures and Tables

**Table 1:** General characteristics of 20 chloroplast genomes in Dipsacales.

Family	Species	Genebank	Size (bp)	LSC (bp)	SSC (bp)	IR (bp)	Total GC (%)	Total Genes	Protein Genes	tRNA Genes	rRNA Genes
Morinaceae	*Morina longifolia*	NC045046	157,149	90,292	18,679	24,089	38.60	128	83	37	8
	*Acanthocalyx alba*	NC045055	157,999	90,750	19,413	23,918	38.36	128	83	37	8
Dipsacaceae	*Triplostegia glandulifera*	NC045051	157,560	90,239	21,099	23,111	37.93	128	83	37	8
	*Dipsacus japonicus*	MN524645	154,709	88,605	18,538	23,783	38.99	128	83	37	8
Adoxaceae	*Adoxa moschatellina*	NC034792	157,238	86,340	18,674	26,112	37.79	128	81	39	8
	*Sinadoxa corydalifolia*	NC032040	157,074	86,430	18,668	25,988	37.76	131	83	40	8
	*Sambucus williamsii*	NC033878	158,305	86,810	18,933	26,281	37.93	132	84	40	8
	*Viburnum odoratissimum*	MN894600	158,757	87,350	18,409	26,499	38.10	132	87	37	8
	*Viburnum dilatatum*	MW346666	158,392	87,070	18,242	26,540	38.10	130	83	39	8
Valerianaceae	*Patrinia scabra*	NC045048	151,267	87,269	18,020	22,989	38.58	128	83	37	8
	*Valeriana officinalis*	NC045052	151,505	87,619	16,288	23,799	38.45	128	83	37	8
Caprifoliaceae	*Triosteum pinnatifidum*	NC037952	154,896	89,007	20,543	22,673	38.54	128	83	37	8
	*Triosteum himalayanum*	NC045219	154,579	89,157	18,682	23,370	38.38	132	86	38	8
	*Triosteum sinuatum*	MW526077	157,178	90,758	18,656	23,882	38.31	135	86	41	8
	*Lonicera ferdinandi*	NC040963	154,513	88,554	18,589	23,685	38.44	128	83	37	8
Linnaeaceae	*Linnaea borealis*	MN548092	161,576	85,609	17,547	29,210	38.26	134	89	37	8
	*Zabelia dielsii*	NC046599	155,584	89,652	19,062	23,435	38.39	128	83	37	8
	*Dipelta yunnanensis*	NC042201	155,359	89,467	19,098	23,397	38.45	128	83	37	8
	*Dipelta floribunda*	NC037955	155,370	89,431	19,052	23,378	38.38	128	83	37	8
Diervillaceae	*Weigela florida*	MN524626	157,966	90,144	21,486	23,168	38.01	128	83	37	8

Abbreviations: GC, GC content.

**Table 2:** List of genes within chloroplast genomes of 20 species.

Category	Group of Genes	Name of Genes
Transcription and Translation	Large subunit of ribosome	*rpl33*, *rpl20*, *rpl36*, *rpl14*, *rpl16 ^a^*, *rpl22*, *rpl2 ^a^*, *rpl23*, *rpl32*
	Small subunit of ribosome	*rps16*, *rps2*, *rps14*, *rps4*, *rps18*, *rps12 ^b^*^,^*, *rps11*, *rps8*, *rps3*, *rps19*, rps7*, *rps15*
	RNA polymerase	*rpoC2*, *rpoC1 ^a^*, *rpoB*, *rpoA*
	Ribosomal RNAs	rrn16*, rrn23*, rrn4.5*, rrn5*
	Transfer RNAs	*trnH-GUG*, *trnK-UUU ^a^*, *trnQ-UUG*, *trnS-GCU*, *trnG-GCC ^a^*, *trnR-UCU*, *trnC-GCA*, *trnD-GUC*, *trnY-GUA*, *trnE-UUC*, *trnT-GGU*, *trnS-UGA*, *trnG-UCC*, *trnfM-CAU*, *trnS-GGA*, *trnT-UGU*, *trnL-UAAa*, *trnF-GAA*, *trnV-UAC ^a^*^,^*, *trnM-CAU*, *trnW-CCA*, *trnP-UGG*, trnI-CAU *, trnL-CAA *, trnV-GAC *, trnI-GAU *, trnA-UGC *, trnR-ACG *, trnN-GUU *, *trnL-UAG*
Photosynthesis	Photosystem I	*psaB*, *psaA*, *psaI*, *psaJ*, *psaC*
	Photosystem II	*psbA*, *psbK*, *psbI*, *psbM*, *psbD*, *psbC*, *psbZ*, *psbJ*, *psbL*, *psbF*, *psbE*, *psbB*, *psbT*, *psbN*, *psbH*
	NADH dehydrogenase	*ndhJ*, *ndhK*, *ndhC*, *ndhB ^a^*^,^*, *ndhF*, *ndhD*, *ndhE*, *ndhG*, *ndhI*, *ndhA ^a^*, *ndhH*
	Cytochrome b6/f complex	*petN*, *petA*, *petL*, *petG*, *petB ^a^*, *petD ^a^*
	ATP synthase	*atpA*, *atpF ^a^*, *atpH*, *atpI*, *atpE*, *atpB*
	Rubisco large subunit	*rbcL*
ATP-dependent protease subunit *p*		*clpP ^a^*
Other genes	Chloroplast envelope membrane protein	*cemA*
	Maturase	*matK*
	c-type	*ccsA*
	Subunit Acetyl- CoA-Carboxylate	*accD*
Genes of unknown function	Conserved ORFs	*ycf1*, ycf2 *, *ycf3*, *ycf4*

^a^ Gene with one intron; ^b^ gene with two introns; * gene with copies.

## Data Availability

The chloroplast genome sequences of *Triosteum sinuatum* were submitted to GenBank (MW526077).

## Supplementary Materials

The following supporting information can be downloaded at: https://www.mdpi.com/article/10.3390/genes13050933/s1, Figure S1: The chloroplast genome of 26 species were compared using the mVISTA program; Figure S2: Analysis of simple sequence repeat (SSR) in twenty-six cp genomes; Figure S3: Codon content of 20 amino acid and stop codons in all protein-coding genes of the twenty cp genomes.
