# Supplementary material for: Complete Chloroplast Genome Sequence of Triosteum sinuatum, Insights into Comparative Chloroplast Genomics, Divergence Time Estimation and Phylogenetic Relationships among Dipsacales
Source: Genes (Basel). 2022 May 23;13(5):933. doi: 10.3390/genes13050933 (PMC9141360; doi:10.3390/genes13050933)
Supplement: Supplementary file 1 [file genes-13-00933-s001.zip › Title of Figures S1-S3.pdf]

Figure S1. The chloroplast genome of 26 species were compared using the mVISTA program. The percentage of sequence identities were evaluated and visual.The direction of arrows represent the direction of genes.Use various color to distinguish genome regions.The purple areas are exons, blue areas are UTR (untranslated regions), red ares are CNS (conserved non-coding sequences).

Figure S2. Analysis of simple sequence repeat (SSR) in twenty-six cp genomes. (a) Numbers different SSRs type detected in twenty-six cp genomes. (b) Frequency of SSR motifs in different repeat types of Triosteum sinuatum Maxim. cp genomes. (c) Frequency of identified SSR in LSC, SSC, and IR regions. (d) Frequency of identified SSR in IGS, CDS, and intron.

Figure S3. Codon content of 20 amino acid and stop codons in all protein-coding genes of the twenty cp genomes. The histogram from the left-hand side of each amino acid shows codon usage within Dipsacales.(From left to right in Figure S3a is *Viburnum dilatatum* Thunb, *Viburnum odoratissimum, Weigela florida, Dipelta floribunda ,Dipelta yunnanensis, Zabelia dielsii, Linnaea borealis;* From left to right in Figure S3b is *Lonicera ferdinandi, Triosteum sinuatum, riosteum himalayanum, Triosteum pinnatifidum, Valeriana officinalis ,Patrinia scabra, Sambucus williamsii;* From left to right in Figure S3c is *Sinadoxa corydalifolia, Adoxa moschatellina, Dipsacus japonicus, Triplostegia glandulifera, Acanthocalyx alba, Morina longifolia)*
