# Supplementary figures and images for: Complete Chloroplast Genome Sequence of Triosteum sinuatum, Insights into Comparative Chloroplast Genomics, Divergence Time Estimation and Phylogenetic Relationships among Dipsacales
Source: Genes (Basel). 2022 May 23;13(5):933. doi: 10.3390/genes13050933 (PMC9141360; doi:10.3390/genes13050933)

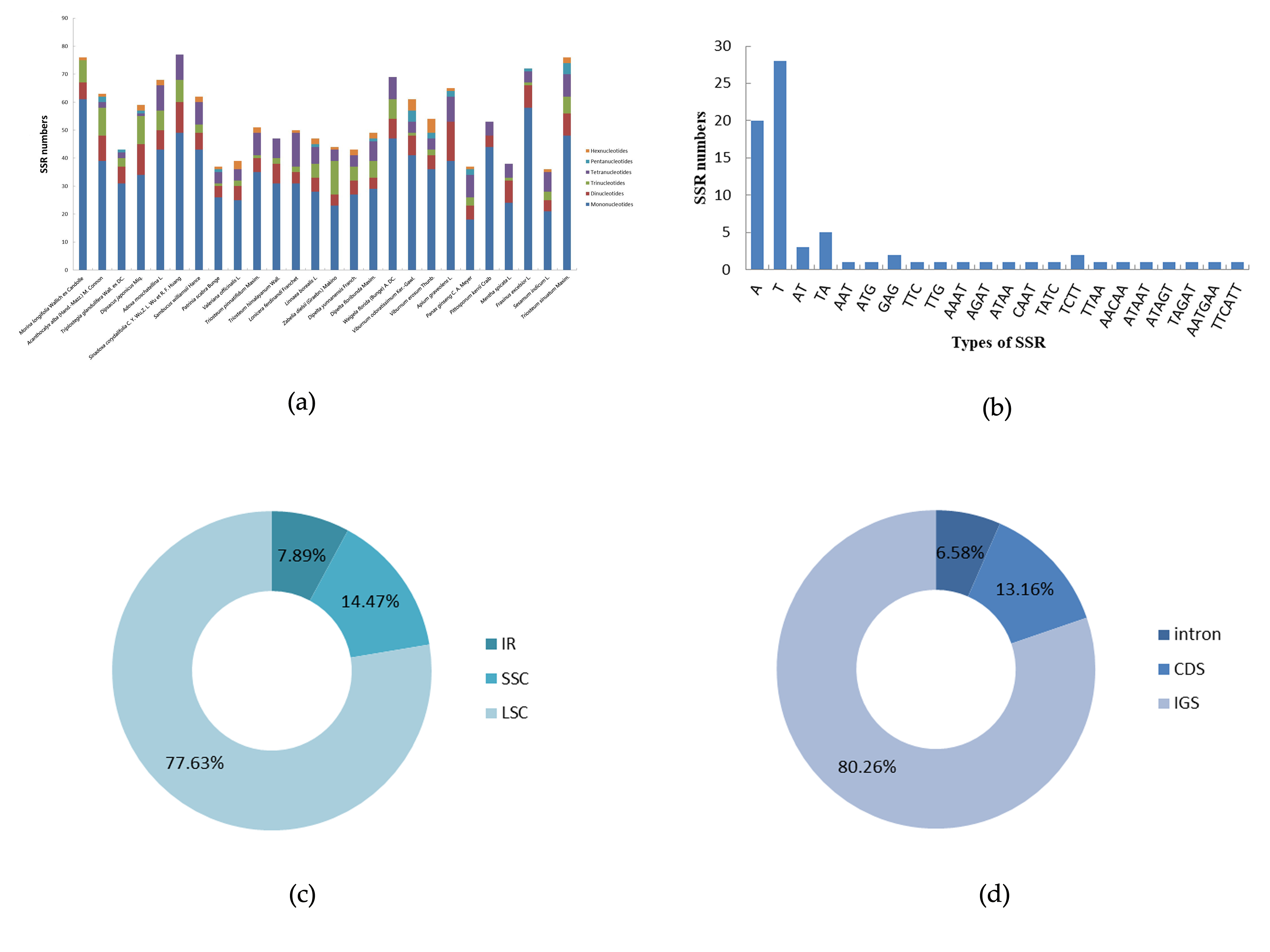

Supplement: Supplementary file 1 [file genes-13-00933-s001.zip › Figure S2.png]
